# dcVar: a method for identifying common variants that modulate differential correlation structures in gene expression data

**DOI:** 10.3389/fgene.2015.00312

**Published:** 2015-10-19

**Authors:** Caleb A. Lareau, Bill C. White, Courtney G. Montgomery, Brett A. McKinney

**Affiliations:** ^1^Tandy School of Computer Science – Department of Mathematics, University of TulsaTulsa, OK, USA; ^2^Arthritis and Clinical Immunology Research Program, Oklahoma Medical Research FoundationOklahoma City, OK, USA; ^3^Laureate Institute for Brain ResearchTulsa, OK, USA

**Keywords:** eQTL, molecular phenotype, genome-wide association study, microarray gene expression, common variant, RNA-Seq

## Abstract

Recent studies have implicated the role of differential co-expression or correlation structure in gene expression data to help explain phenotypic differences. However, few attempts have been made to characterize the function of variants based on their role in regulating differential co-expression. Here, we describe a statistical methodology that identifies pairs of transcripts that display differential correlation structure conditioned on genotypes of variants that regulate co-expression. Additionally, we present a user-friendly, computationally efficient tool, dcVar, that can be applied to expression quantitative trait loci (eQTL) or RNA-Seq datasets to infer differential co-expression variants (dcVars). We apply dcVar to the HapMap3 eQTL dataset and demonstrate the utility of this methodology at uncovering novel function of variants of interest with examples from a height genome-wide association and cancer drug resistance. We provide evidence that differential correlation structure is a valuable intermediate molecular phenotype for further characterizing the function of variants identified in GWAS and related studies.

## Introduction

Recent advances in DNA and RNA sequencing technology have afforded the ability to interrogate variance in gene expression and allele frequency distributions to determine associations with phenotypic variance, including disease. However, many of the variants uncovered in the genome-wide association study (GWAS) era have yielded few functional characterizations for particular phenotypic associations (Edwards et al., 2013). Moreover, as only 5% of GWAS associations code amino acid changes in corresponding protein structures, the elucidation of the genetic architecture leading to phenotypic variance has been broadened to search for variants that differentially affect transcription called expression quantitative trait loci (eQTLs; Majewski and Pastinen, 2011). Though the increased examination of eQTLs has further functionalized many variants, the function of a large fraction of association variants has yet to be explained.

In transcriptomic analyses, a strong co-expression or correlation between a pair of expression probes is used to infer a shared biological function or pathway. In these co-expression studies, the correlation between pairs of genes is typically assumed to be uniform across all samples, even in datasets designed to examine phenotypic differences. However, the inter-group (phenotypic) differences in correlated expression data may provide more powerful insights into phenotypic variance than a uniform correlation measure. For example, Zhang et al. (2010) constructed a differential gene co-expression network in microarray data that identified prognostic biomarkers in identifying treatment outcomes for chronic lymphocytic leukemia. In a recent study, we incorporated differential co-expression in a network theory analysis to identify genetic markers associated with phenotypic differences in microarray data (Lareau et al., 2015). Differential co-expression analyses compute the mean pairwise correlation difference between groups, typically defined by the presence or absence of a disease diagnosis (de la Fuente, 2010). While a change in an individual gene’s expression may influence the phenotype in isolation, a more probable explanation is that the change in one gene’s expression will have a cascading interactive effect (Lareau et al., 2015). Aggregating these differential co-expression effects in a network structure, we were able to identify hub effectors in a seasonal influenza vaccine dataset that were well-situated within the known pathways of immune response to vaccination (Lareau et al., 2015). However, our previous statistical approach did not include the effect of genotypic variation on differential co-expression.

In a recent discussion of differential correlation methods, less than a quarter of the methods reviewed computed a significance statistic and failed to address the need to correct for multiple testing (Kayano et al., 2014). Of these discussed methods, almost all were implemented in an *R* package but were not designed for variants to define group inclusion (Liu et al., 2010; Yang et al., 2013). Other reviews of differential co-expression analyses have promoted the use of algorithms that compute the Pearson correlation and Fisher’s *Z*-test due to their simplicity and ability to elucidate statistical relationships (de la Fuente, 2010; Fukushima, 2013). In contrast to Wang et al. (2012), we provide a tool for investigators to perform genome-wide characterization of their QTL datasets, and our implementation of differential co-expression QTL incorporates the above statistical considerations when correcting for multiple hypotheses for differential correlation effects modulated by variants. We use a Bonferroni correction in this study; however, the tool includes an FDR option for users wanting a less stringent correction.

In the present study, we provide a novel computational framework that uncovers variants associated with differential correlation structures in eQTL and RNA-Seq data. Like eQTL analyses, we hypothesize that the use of this methodology to infer differential co-expression variants (dcVars) can potentially functionalize variants identified in GWAS and related association studies. To demonstrate this, we implement our methodology in a computationally efficient tool called dcVar and apply this tool to the HapMap3 eQTL dataset. We discuss two of the significant results that demonstrate added functionality to previously implicated variants in phenotypic variance. Our results suggest that the examination of variants that induce differential correlation structure in expression levels provides a powerful intermediate molecular phenotype that can be readily examined in parallel with existing eQTL as well as RNA-Seq analyses.

## Materials and Methods

### Computing Differential Co-expression

To model differential co-expression of two genes between two groups of subjects separated by the genotype of a variant based on a given genetic model, we use the Fisher’s *Z*-test discussed in the following equations. First, the Pearson correlation of expression *E* is calculated for pairs of genes *i* and *j* for subjects within each of the two genotype groups, *G* = *G_1_* or *G_2_*:

(1) $$r_{ij}^{\left(G\right)}=\frac{\mathrm{cov}\left(E_{i},E_{j}\right)}{\sigma_{E_{i}}\sigma_{E_{j}}}\,$$

The within-group correlation values are then Fisher z-transformed for each group *G_1_* and *G_2_*:

(2) $$z_{ij}^{\left(G\right)}=\frac{1}{2}\,\;In\left|\frac{1+r_{ij}^{\left(G\right)}}{1-r_{ij}^{\left(G\right)}}\right|\,$$

The difference of the z-transformed correlation values between groups *G*_1_ of sample size *m_1_* and *G*_2_ of sample size *m_2_* are computed for genes *i* and *j*:

(3) $$Z_{ij}=\frac{\left|Z_{ij}^{G_{1}}-Z_{ij}^{G_{2}}\right|}{\sqrt{\frac{1}{m_{1}-3}+\frac{1}{m_{2}-3}}}\,$$

The resulting statistic produced by Eq. (3) is assumed to be normally distributed, where the *p*-value associated with the difference in within-group correlation can be determined from the *Z* score (Cohen and Cohen, 1983). More robust alternatives to this approach are described in the conclusions.

In order to separate individuals into groups *G_1_* or *G_2_* on the basis of genotype, we propose three different models. For a hypothetical single nucleotide polymorphism (SNP) with a major allele A and minor allele T, three possible separations are implemented in our algorithm. First, a dominant encoding model where the presence of one or more minor alleles results in individuals with an AA genotype comprising group *G_1_* while group *G_2_* is composed of individuals with either an AT or TT genotype. Conversely, one can impose a recessive model where individuals with either the AA or AT genotype are assigned to *G_1_* whereas only subjects with both minor alleles (TT) constitute group *G_2_*. Finally, we implemented a model denoted “homozygous,” such that group *G_1_* is composed of only subjects with both major alleles (AA) whereas group *G_2_* individuals have both minor alleles (TT). Thus, the heterozygotes for the particular variant (AT) are excluded from the analysis. In the conclusions, we discuss additional genotype encodings that may be combined with a linear model of co-expression.

### Implementation of dcVar

**FIGURE 1:**
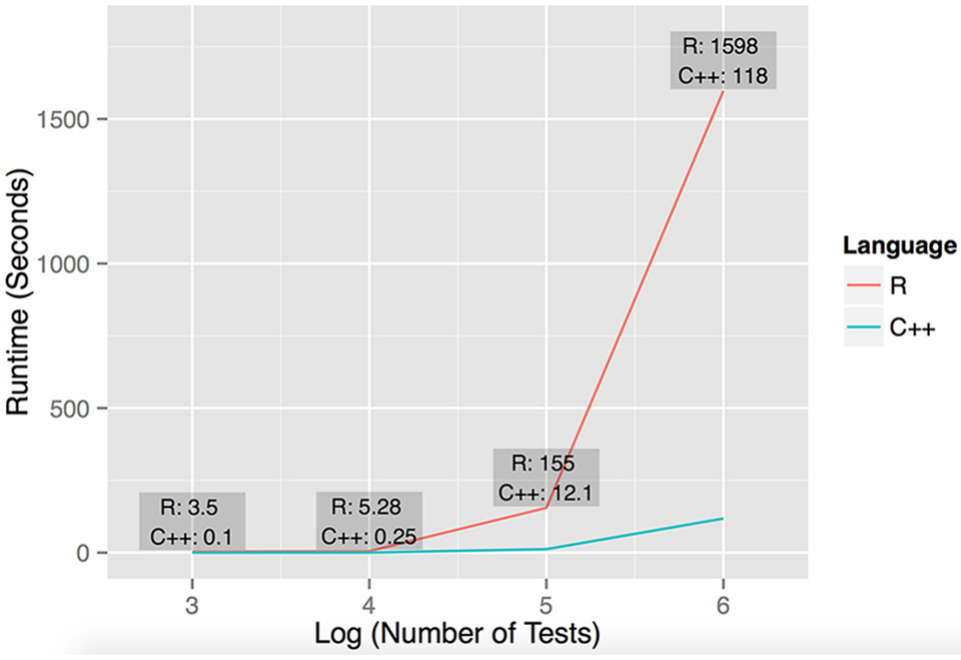
**Performance of R and C++ implementations of dcVar.** Runtime performance was computed executing dcVar on random subsets of the HapMap 3 eQTL data (491 individuals) on a Linux desktop with an AMD FX(tm)-4100 Quad-Core Processor with a 3.6 GHz CPU and 8 GB RAM. The four data points representing the runtime were computed from (1) one variant and 100 expression probes for 10^3^ tests, (2) ten variants and 100 probes for 10^4^ tests, (3) 100 variants and 100 probes for 10^5^ tests, and (4) 100 variants and 1000 expression probes for 10^6^ tests. The average speedup of C++ against R on these four data points was 21x, noting the average speedup on large-scale simulations is closer to 15×. The graph reflects the execution time on the y-axis against the log_10_ of the number of tests performed.

We implemented the described statistical framework as a C++ tool called dcVar based on the PLINK source code (Purcell et al., 2007). To compare the performance of the C++ implementation of dcVar, we also implemented the algorithm in R. The R implementation copies the exact methods (the nested ‘for’ loops) of the C++ code for the comparisons. We note that the R code could be further optimized, for example through parallelization, but our comparison is designed to characterize the difference between straightforward R and C++ implementations. The C++ implementation of our algorithm demonstrates significantly improved performance over R (**Figure 1**), which is an important consideration when developing bioinformatics software for very large data sets (Davis et al., 2011, 2013; Lareau and McKinney, 2015). While some existing tools can compute differential correlation in expression data, dcVar is the first to our knowledge to use SNP variants to define group comparison. Moreover, virtually all tools that uncover differential co-expression are implemented in R (Dawson et al., 2012; Fukushima, 2013), which is markedly slower than our C++ implementation (**Figure 1**). As the computational scale of a dcVar analysis is order (Number of Transcripts^2^ × Number of SNPs), we emphasize the enhanced utility of dcVar over existing tools to efficiently compute differential correlation especially on a genome-wide scale. The C++ and R implementation of dcVar and a tutorial are available for download and installation at http://insilico.utulsa.edu/dcVar.php.

### Application to HapMap3 Data

To assess the utility of the proposed method in terms of biological relevance and computational performance, we applied dcVar to an eQTL dataset of 491 HapMap3 individuals from six different ethnicities (Altshuler et al., 2010). After performing standard quality control on the publicly available data set (2% SNP missingness, 2% individual missingness, Hardy–Weinberg Equilibrium, restricting to autosomal SNPs/transcripts), we applied the default LD pruning option in PLINK (Purcell et al., 2007). Next, we restricted remaining SNPs such that allele frequencies were between 0.2 and 0.8. These preprocessing steps resulted in 70,716 SNPs and 18,392 autosomal transcripts. To adjust for the multiple ethnicities in HapMap3, we performed a quantile normalization of each transcript, which has been previously described (Veyrieras et al., 2008) and implemented in the HapMap and other eQTL datasets (Becker et al., 2012; Banovich et al., 2014). We note that this normalization provides sufficient correction for ancestry in our eQTL dataset (Veyrieras et al., 2008).

The number of transcripts was filtered by selecting the top 10,000 transcripts using a total variance filter after quantile normalization (Veyrieras et al., 2008) was applied. The total matrix of 10,000 transcripts and 70,716 SNPs were analyzed to determine SNPs that significantly predicted differential correlation structures between pairs of transcripts. To determine significant effects, we used the dominant encoding model and pruned results that were not significant after correcting for multiple testing (*p* > 1.41 × 10^-14^), which was computed by dividing 0.05 by the number of tests performed ((10,000/2) × 9,999 × 70,716). Additionally, to estimate the type-I error of dcVar, we permuted the same HapMap3 dataset by randomizing the genotypes for each variant while fixing the expression values for each individual. The same dominant model was applied as described in our regular analysis, and the number of significant probes against our corrected threshold were counted as false positives.

## Results

### Benchmarking and Results of dcVar Implementation

To assess the performance of our R and C++ implementations of dcVar, we analyzed random subsets of the full (491 individuals, 70,716 SNPs, 10,000 transcripts) HapMap3 eQTL dataset using the dominant encoding model. **Figure 1** shows the benchmarking results of executing dcVar on a Linux desktop with an AMD FX(tm)-4100 Quad-Core Processor with a 3.6 GHz CPU and 8 GB RAM. Of the four example benchmarks computed, the C++ version of dcVar outperformed an identical R implementation by an average of 21× or 15× in scenarios with larger numbers of tests. The order of magnitude difference in performance between these implementations becomes more pronounced as the number of tests approaches a genome-wide analysis (**Figure 1**). Our implementation of a framework to compute differential correlation from variants in C++ provides a tool for researchers to uncover these effects in a genome-wide eQTL or RNA-Seq analysis. While benchmarking was performed on a desktop machine, the analysis performed on the HapMap dataset was executed on a computing cluster available at Oklahoma Medical Research Foundation. We note that similar genome-wide analyses using dcVar would be best performed on a similar local computing cluster or through the NIH biowulf cluster.

### Case 1: GWAS Hit Characterization

While many GWAS analyses have successfully identified SNPs associated with a variety of diseases and quantitative phenotypes, the implicated variants are often difficult to situate in a functional context (Edwards et al., 2013). Even if associations are validated by the biological mechanism of the coding gene, demonstrable functional effects of the SNP have been rarely characterized (Edwards et al., 2013). Consequently, the use of eQTLs to elucidate the molecular consequences of DNA variants has become a popular method to functionalize GWAS hits (Majewski and Pastinen, 2011; Edwards et al., 2013). However, many GWAS associations lack any characterization of molecular effects even after dozens of eQTL analyses. For example, rs823094, a variant located in the gene nuclear ubiquitous casein and cyclin-dependent kinases substrate (*NUCKS1*), has previously been associated with pubertal height growth (Cousminer et al., 2013). As *NUCKS1* is involved in the proliferation of several growth factors (Ilaria and Van Etten, 1995; D’Andrea et al., 1998), an association with a variant in *NUCKS1* with pubertal height growth is supported biologically. However, like most other implicated SNPs in GWAS, no functional effect of rs823094 has been determined for the associated phenotype.

**FIGURE 2:**
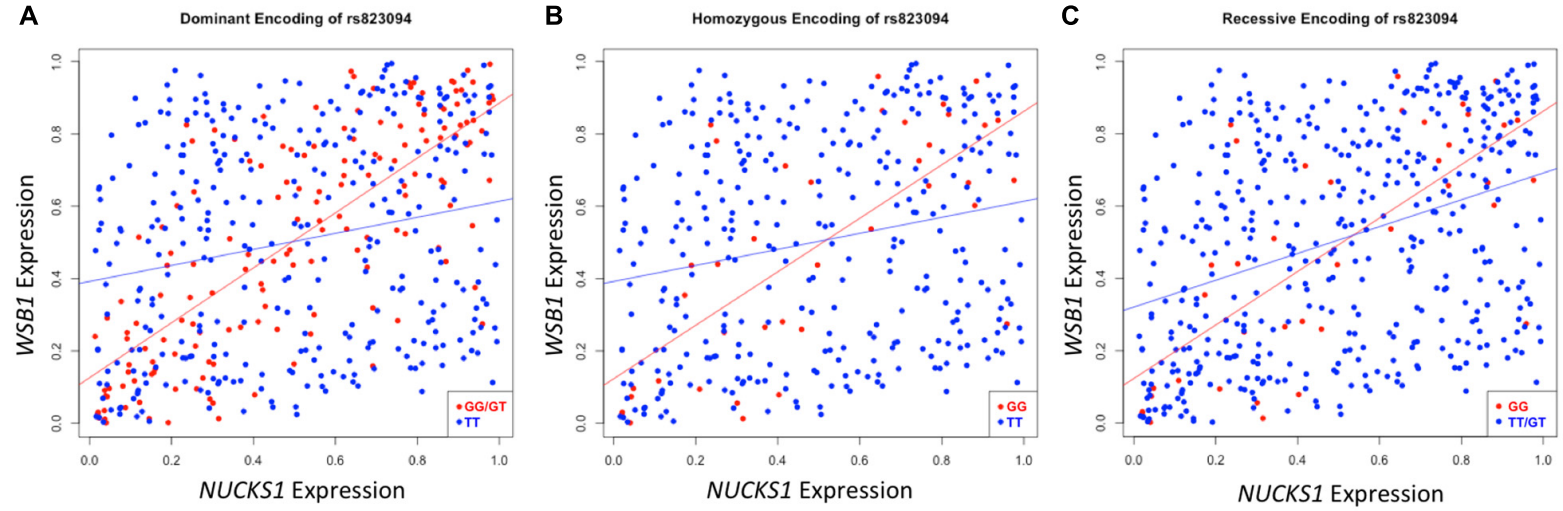
**Differential correlation of *NUCKS1* and *WSB1* by rs823094.** The plots represent differential correlation structures under all three models, dominant **(A)**, homozygous **(B)**, and recessive **(C)**, where T is the major allele and G the minor for rs823094, a variant located in the intron of *NUCKS1*. Each encoding yields a statistically significant difference in correlation under each group separator (*p*_dom_ = 1.69 × 10^-15^; *p*_hom_ = 0.00012; *p*_rec_ = 0.0031). Both *NUCKS1* and *WSB1* have been implicated in pathways leading to growth while rs823094 has been associated with phenotypic variance in height.

After executing dcVar on our HapMap eQTL dataset, we observed a significant differential correlation structure between this height-associated variant, rs823094, its corresponding gene, *NUCKS1*, and the WD repeat and SOCS box containing 1 (*WSB1*) gene (*p*_dom_ = 1.69 × 10^-15^). As *WSB1* modulates thyroid hormone activation and parathyroid hormone-related peptide secretion in developing growth plate (Dentice et al., 2005), a common biological consequence of these two genes (growth) helps explain the observed association with the variant and the phenotype (height). Differential correlation remains statistically significant (**Figure 2**) under all three models for stratifying groups: dominant (A), homozygous (B), and recessive (C). Like eQTL analyses that use transcriptomic markers to bridge the gap between SNP associations and phenotypic variance, dcVar uncovers additional transcriptomic markers that interact in the functional network.

**FIGURE 3:**
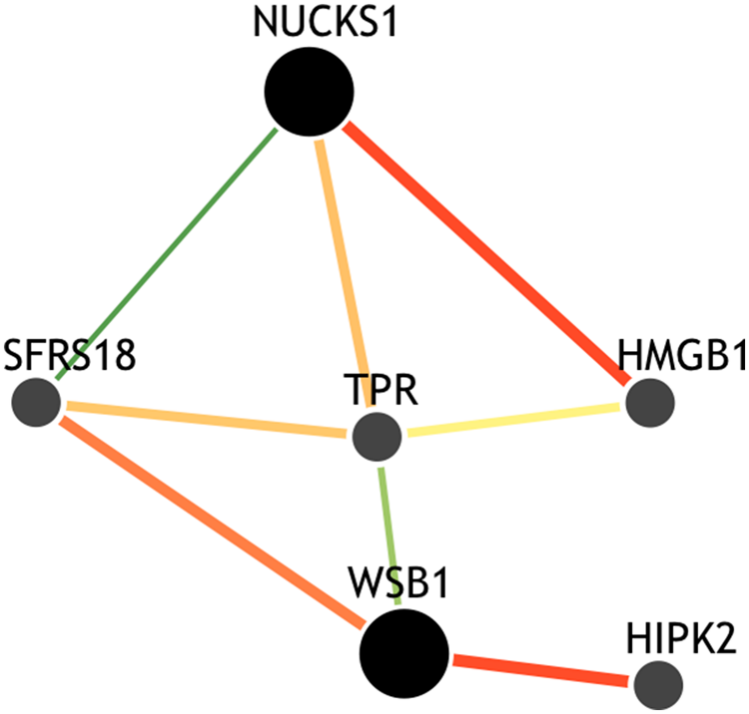
**Gene interaction network of *NUCKS1* and *WSB1*.** The network was created using the IMP Server (25) using a minimum edge confidence of 0.25 and including four additional predicted genes. The network predicts an intermediate interaction between *NUCKS1* and *WSB1* separated by alternative paths between *SFRS18* and *TPR* genes, suggesting a potential mechanism for this differential correlation effect.

**FIGURE 4:**
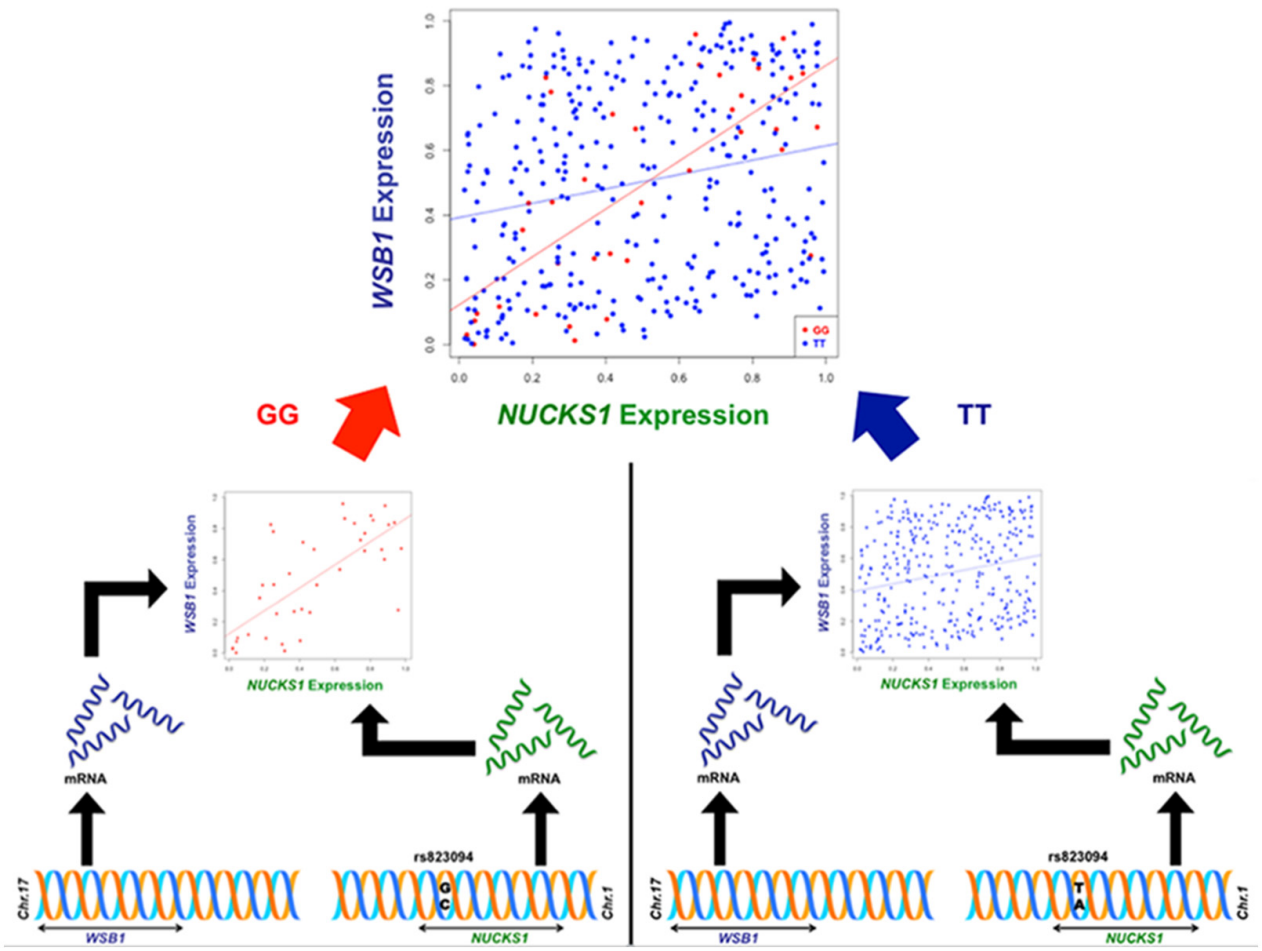
**Illustration of differential correlation mechanism for *NUCKS1* and *WSB1*.** The left panel shows the generation of a correlation structure conditioned on the minor allele (G, red) in rs823094, a variant in the intron of *NUCKS1*, while the right lacks a discernable correlation structure when conditioned on the major allele (T, blue). The difference in correlation between *NUCKS1* and *WSB1* from individuals with two minor alleles (GG, red) and individuals with two major alleles (TT, blue) was statistically significant (adjusted *p*_hom_ = 0.00012).

Though the variant modulating the differential correlation in this example is encoded in an intron in *NUCKS1*, which has a similar biological role as *WSB1* in promoting growth, a direct interaction between the products of these genes has not been observed. However, we note two pieces of evidence that point to a plausible connection between *NUCKS1* and *WSB1* and a possible biological explanation for the genotype-specific differential correlation structure observed. First, a remarkable number (107) of transcription factors (45.5% of factors in EncodeQT) regulate both of these genes within 500 bp of their transcription start sites as determined by ChIP-Seq signals generated from the ENCODE Project (Auerbach et al., 2013). As these two genes are joined in transcriptional activity through these factors, a differential expression effect as we identified could amplify the differential correlation signal in the relative expression values. Additionally, using the Integrated Multi-species Prediction (IMP) server (Wong et al., 2012), we note the modulation of interactions between *NUCKS1* and *WSB1* by two genes, *SFRS18* (splicing factor, arginine/serine-rich 18) and *TPR* (translocated promoter region), as shown in **Figure 3**. While a specific mechanism of activity leading to phenotypic variance in height is unclear, the likely interaction between *NUCKS1* and *WSB1* through a degree of separation as predicted by IMP suggests a high level biological interaction. Thus, at both the regulatory level via transcription factors and the interaction level via intermediate genes, some interaction exists between *NUCKS1* and *WSB1*, demonstrating the plausibility of a differential correlation existing between these two genes that better characterizes the association of rs823094. We illustrate this differential correlation process using the homozygous model (**Figure 4**). The left (**Figure 4**) depicts the emergence of a strong correlation structure (*r* = 0.715) for individuals with a GG genotype for rs823094, and the right panel shows a weaker correlation (*r* = 0.219) for those with both major alleles, noting that a strong correlation (*r* = 0.775) is present for the heterozygotes (GT genotype).

### Case 2: Characterizing Known Interactive Effects

**FIGURE 5:**
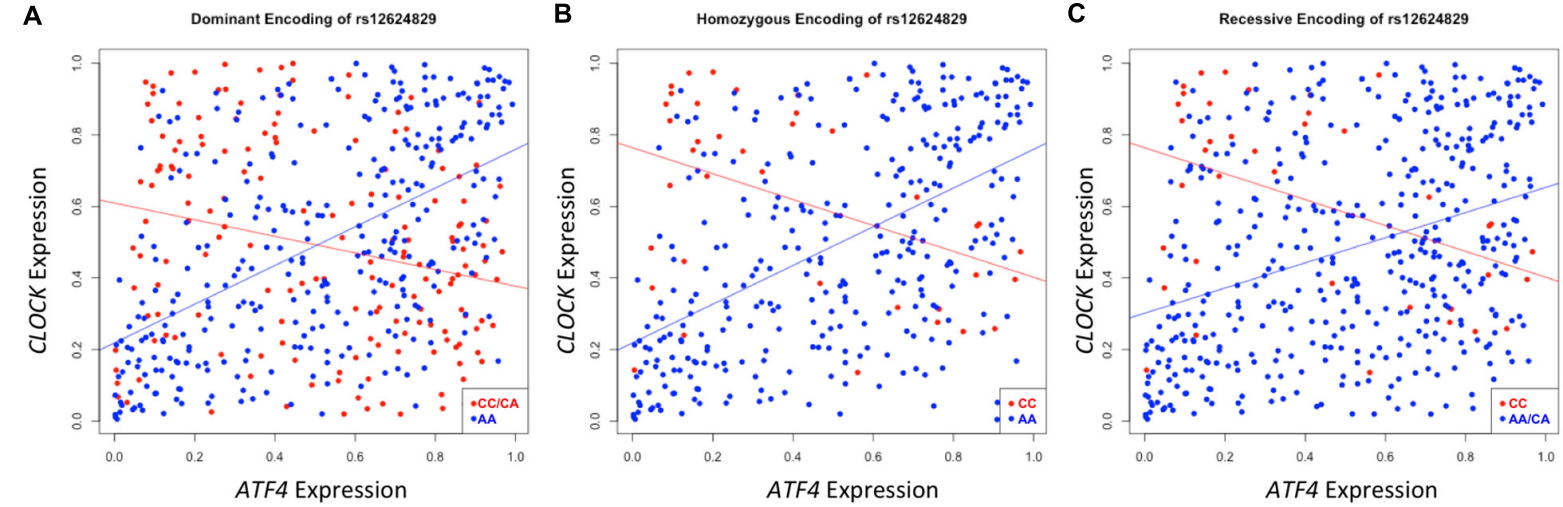
**Differential correlation of *ATF4* and *CLOCK* by rs12624829.** Each plot represents differential correlation conditioned by different models: dominant **(A)**, homozygous **(B)**, and recessive **(C)**, where allele A is the major and C is the minor in rs12624829, a variant located in introns in a gene-dense region of chromosome 20 shown to affect the binding of *MAX*. Each encoding yields a statistically significant difference in correlation under each group separator (*p*_dom_ = 8.63 × 10^-19^; *p*_hom_ = 3.65 × 10^-9^; *p*_rec_ = 2.91 × 10^-6^). *ATF4*, *CLOCK*, and *MAX*, a transcription factor whose binding is differentially affected by rs12624829, have been implicated in differential drug response in certain types of cancers.

A second instance of significant differential correlation was observed between the activating transcription factor 4 (*ATF4*) and circadian locomotor output cycles kaput (*CLOCK*) genes regulated by rs12624829 (*p*_dom_ = 8.25 × 10^-19^). As the protein interactions between these two genes has been well-characterized in the transcription regulation system, the *CLOCK* and *ATF4* interaction has also been implicated in regulating drug resistance in the A549 cancer cells (Igarashi et al., 2007). However, the exact mechanism of action between these two genes and how their interaction modulates systematic differential response to cancer drugs remains unclear (Igarashi et al., 2007). Strong correlation exists between *CLOCK* and *ATF4* in individuals with the major alleles for rs12624829 (**Figure 5**). However, the minor alleles in this variant cause this correlation to decrease. As the synergistic effect of *CLOCK* and *ATF4* has been implicated in multidrug resistance through glutathione-dependent redox system, a breakdown in this correlation could lead to differential drug response in human cancer (Igarashi et al., 2007).

While the implicated SNP, rs12624829, represents a trans-effect to the two implicated probes, this variant affects the binding of a transcription factor, myc-associated factor X (*MAX*), in the same A549 cell line (Boyle et al., 2012). Interestingly, *MAX* also binds to both *CLOCK* and *ATF4* near the transcription start sites to regulate the expression of each gene (Auerbach et al., 2013). Although altering the binding of this transcription factor could lead to differential drug response in cancer cell lines through the downstream effect of *CLOCK* and *ATF4*, we note that *MAX* itself has been implicated in cancer drug resistance as a prior study used the factor to distinguish cancer subtypes that explained differential drug response (Heiser et al., 2012). Taken together, each of the identified factors has been implicated in differential drug response in cancer cell lines. However, the identification of differential correlation structure provides the first integrated hypothesis that suggests *MAX* could modulate the co-expression of *ATF4* and *CLOCK*, leading to differential cancer drug susceptibility.

### Analysis of HapMap3 Results

Using the dominant model, our methodology computed a significant differential correlation structure for 47.1% of the 10,000 expression probes and 13.7% of the 70,716 common variants included for analysis using the *p* < 1.41 ×10^-14^ threshold. These results represent 3,223,172 SNP × gene × gene trios (representing 0.00009% of computed interactions) and 1,237,788 unique gene pairs. These relatively high percentages likely indicate inflated statistics for a few reasons. First, variants in strong LD that fall outside the 50 kb region from the PLINK LD pruning flag would inflate the number of observations while representing only one true effect. In addition, transcripts that exhibit strong covariance would also inflate the number of positive associations.

The two cases highlighted in the manuscript were selected based on the strength of the association (1–4 orders of magnitude greater significance than the threshold) and biological relevance. In particular, we used two approaches to prioritize the hits using biological knowledge. First, we annotated the pairs of gene expression probes with interaction scores from STRING-DB. The ATF4-CLOCK example represented 1 of 180 gene pairs that had a STRING interaction score >800. Second, we enriched 40 SNPs for known genome-wide association terms from general population phenotypes (e.g., height) using the NHGRI database. The NUCKS1-WSB1 variant (rs823094) represented one of 40 of these enriched variants. Additionally, we note that other multiple phenotype regression models did not uncover these effects identified by dcVar. Notably, the two examples that we highlight in this manuscript were not significant using the Plink.multivariate association model (NUCKS1/WSB1: *p* = 0.9969; ATF4/CLOCK: *p* = 0.1826) (Ferreira and Purcell, 2009). Though other significant effects could have been characterized in the manuscript, these would require external validation of the differential correlation association.

### Comparison to eQTL Discovery

As dcVar computes the effect of variant alleles on pairs of expression values for genes through differential correlation, we sought to determine if these effects were captured under standard linear regression as used in most eQTL analyses. Using a nominal *p* < 0.05 significance threshold, only 1.84% of SNP-gene pairs significant in our dcVar computation were significant. When further requiring both gene expression probes to be significant for the SNP × gene × gene trios uncovered by dcVar, only 0.29% of our uncovered effects were significant in standard eQTL models. In the two cases highlighted above, the univariant eQTLs were not significant for either rs823094 (*p* = 0.9959 for NUCKS1; *p* = 0.9408 for WSB1) or rs12624829 (*p* = 0.2322 for ATF4; *p* = 0.3089 for CLOCK). As eQTL studies often employ more stringent *p*-value thresholds for discovery, few significant results uncovered by the dcVar approach would be represented in a typical eQTL computation.

### Estimation of Type-I Error

To estimate the false positive rate (FPR) of dcVar, we performed a permutation-based analysis on the HapMap3 eQTL data. While maintaining the order of the 10,000 expression probes, we permuted the genotypes of a given variant and applied dcVar to test differential co-expression for all pairs of probes. We repeated this for all 70,716 SNPs that passed the pruning process. When we use the Bonferroni threshold based on all SNP-probe-probe trios (0.05/(70,716^∗^10,000^∗^9,999/2) = 1.41 × 10^-14^), only a single trio in the permutation simulation survived the threshold. We used this strict threshold in the current application of dcVar to the HapMap3 data. As a side note, if one were to use a per-variant corrected threshold of 1x10^-9^ (from 0.05/(10,000^∗^9,999/2)), 15,699 SNP-probe-probe trio statistical tests would be significant in the permuted data.

## Discussion

The methodology described is designed to uncover variants whose minor alleles affect the correlation structures in expression data. This can be manifested in at least two ways. First, as with case 1, minor alleles could generate correlation through some modulated interaction between two transcripts. The resulting synergistic effect of the modified correlation structure could amplify a genetic effect, in turn increasing the phenotypic effect. For example, we suggest that the effect of a correlation structure of two genes, *NUCKS1* and *WSB1*, each of which has been previously implicated in growth, could amplify the individual effects of the genes, leading to observable phenotypic variance in height. Conversely, minor alleles can disrupt the correlation structure of genes, as seen in case 2. In this example, we suggest that a perturbed correlation structure of *ATF4* and *CLOCK* could be the result of differential activity of the *MAX* transcription factor. The differential correlation mechanism unites multiple observations of these three factors leading to differential drug resistance in certain types of cancer. Ultimately, additional phenotypic data in more specific eQTL datasets or molecular experiments will be required to validate these hypotheses. Nevertheless, the potential functional characterization of these variants and expression levels using available data demonstrates the added benefit of performing dcVar analysis alongside existing analyses. We note that other methods for computing genotype-specific differential correlation did not uncover the effects above (Wang et al., 2012).

Though we applied dcVar to an eQTL dataset composed of microarray and genotypes, this methodology could be used to interpret results from RNA-Seq experiments. As non-coding RNA have been implicated as genetic determining elements of complex phenotypes (Majewski and Pastinen, 2011), we assert that a plausible mechanism for non-coding RNA activity is through nucleic acid interactions influencing correlation structures, and this activity could be modulated by variants detected in RNA-Seq data. The dcVar tool described herein can be applied to uncover these effects, noting the sample imbalances in rare variants when computing differential correlation and the differences in detecting transcript variation between RNA-Seq and microarray technology (Ballouz et al., 2015).

As our implementation of differential co-expression uses a dichotomization of the genotype, we implemented three models — dominant, recessive, and homozygous — to compute differential correlation modulated by variants. While dcVar can efficiently compute each model in a genome-wide analysis, we used the dominant model to best balance the sample sizes between the two groups being compared. As differential correlation has been applied previously to case/control microarray data with roughly equal sample sizes (Lareau et al., 2015), the use of a dominant model and a high minor allele frequency cutoff (20%) best balances the sample sizes in the two groups. We note that the effects observed in our two highlighted cases were retained in the recessive and homozygous model when using the dominant model for discovery. While we recommend the use of the dominant model, subsequent analysis of positive results using the recessive and homozygous models could elucidate the possible dominance, recessive, or dosage behavior of the minor allele. Thus, we suggest the type of model employed should be carefully considered by the user through knowledge of the test at hand.

The computational efficiency of dcVar enables users to incorporate information from variants that modulate differential correlation structure through a variety of means. In the analysis described in this study, we tested our approach on the HapMap eQTL data, so there was not an *a priori* phenotype or disease association to provide targets for functional characterization. However, we hypothesized that this data would contain dcVar effects that would provide functional information for many phenotypic and disease associations. Thus, we considered thousands of transcripts and variants genome-wide followed by prioritization using biological knowledge from GWAS studies, the STRING database, and the IMP server. A user with non-coding disease associations from a GWAS may benefit from a systematic integration of dcVar statistics with these other sources of biological knowledge, for example, in a Bayesian framework. From the pipeline used in the current study, we chose to focus on dcVar effects on height because it is a universal phenotype and on cancer drug resistance because of the potential implications in pharmacogenomics. For other phenotypes, a specific association from a GWAS may be involved in a dcVar effect as the genotype, as one of the differentially co-expressed transcripts, or downstream of another dcVar in the wider co-expression network. An integrative network model of SNPs, expression, co-expression, and differential co-expression effects may greatly improve our understanding of biological mechanisms in the genome. Moreover, from our characterization of the two effects described in detail, we note that researchers seeking to functionalize a particular variant or gene with a variety of quantitative data (e.g., gene expression, ChIP-Seq, epigenetic methylation, etc.) may do so with the dcVar tool. Future applications of dcVar to genome-wide eQTL datasets will further characterize the highly interconnected network of possible regulatory variants involving the dcVar intermediate molecular phenotype.

A limitation of our approach is the modeling of changes in co-expression between subjects conditioned on only two genotype groups. Specifically, we compute the differential co-expression between subjects grouped by a recessive or dominant encoding, which may not properly model the co-expression variation in the heterozygous group. A potentially more powerful approach would be to estimate a linear model of the co-expression using an additive encoding, which has been more popular in most GWAS and other genomics studies (Cantor et al., 2010), although this may have less power when the causal model is recessive. A co-dominant model has been shown to have good overall performance for simulations of a variety of inheritance models (Lettre et al., 2007).

Another limitation of the dcVar approach is the potential for increased type-II error. In order to limit type-I errors associated with the very large number of dcVar hypotheses, we used a strict Bonferroni correction. The dcVar tool includes a Benjamini–Hochberg FDR option, which gives the user one way to decrease the type-I error. Our use of Pearson correlation to compute co-expression in genotype groups also increases the risk of type-II error and sensitivity to outliers. More robust statistics such as Spearman and bootstrapping methods may decrease sensitivity to outliers and deviations from normality (Fisher’s z-transform notwithstanding) and thereby decrease type-II error. Future studies are needed to demonstrate the extent to which robust statistics may improve dcVar effect detection. Finally, we note that our previous study employing differential co-expression between case-control status identified hub determinants of phenotypic variation by integrating information from the broader differential co-expression network (Lareau et al., 2015). While the current study provides the first computationally feasible tool to uncover individual variants that lead to differential correlation genome-wide, the aggregation of multiple interactive effects between probes and variants could further uncover functional associations. Expanding dcVar to include network structure may further elucidate the function of variants that affect differential co-expression and phenotypic variation.

## Author Contributions

CL and BW wrote the software and analyzed the data. CL, CM, and BM wrote the manuscript. All authors have read and approved of the final version of the draft.

## Conflict of Interest Statement

The authors declare that the research was conducted in the absence of any commercial or financial relationships that could be construed as a potential conflict of interest.
